# Is Mossy Fiber Sprouting a Potential Therapeutic Target for Epilepsy?

**DOI:** 10.3389/fneur.2018.01023

**Published:** 2018-11-30

**Authors:** Clarissa F. Cavarsan, Jackeline Malheiros, Clement Hamani, Imad Najm, Luciene Covolan

**Affiliations:** ^1^Department of Physiology, Universidade Federal de São Paulo, São Paulo, Brazil; ^2^Harquail Centre for Neuromodulation, Sunnybrook Health Sciences Centre, University of Toronto, Toronto, ON, Canada; ^3^Epilepsy Center, Neurological Institute, Cleveland Clinic, Cleveland, OH, United States

**Keywords:** temporal lobe epilepsy, mossy fibers sprouting, epileptogenesis, deep brain stimulation, hippocampus

## Abstract

Mesial temporal lobe epilepsy (MTLE) caused by hippocampal sclerosis is one of the most frequent focal epilepsies in adults. It is characterized by focal seizures that begin in the hippocampus, sometimes spread to the insulo-perisylvian regions and may progress to secondary generalized seizures. Morphological alterations in hippocampal sclerosis are well defined. Among them, hippocampal sclerosis is characterized by prominent cell loss in the hilus and CA1, and abnormal mossy fiber sprouting (granular cell axons) into the dentate gyrus inner molecular layer. In this review, we highlight the role of mossy fiber sprouting in seizure generation and hippocampal excitability and discuss the response of alternative treatment strategies in terms of MFS and spontaneous recurrent seizures in models of TLE (temporal lobe epilepsy).

## Introduction

Epilepsy is one of the most frequent neurological disorders, affecting more than 60 million people worldwide (1). It is a complex and disabling disease, with no cure and often without effective treatment (2). Current drug therapies are still not able to stop the disease or inhibit its development, but merely control convulsive seizures (3). The clinical hallmark of epilepsy is the occurrence of spontaneous recurrent seizures. It is estimated that up to 50% of all cases are triggered by “initial precipitating injuries,” such as status epilepticus (SE), stroke, and traumatic brain injury (TBI) (4, 5). Despite the huge number of preclinical and clinical studies, there is still a limited understanding of the basic mechanisms underlying epilepsy development or “epileptogenesis” (3).

Current epilepsy therapies rely on symptomatic strategies, either pharmacological or surgical, both aiming to suppress seizures, but do not target epileptogenesis (6). As the disease progresses, up to 30% of patients with epilepsy, particularly those with mesial temporal lobe epilepsy (MTLE) caused by hippocampal sclerosis, become pharmacoresistant, exhibiting medically intractable recurrent seizures (2). These patients are often amenable to surgical removal of the sclerotic structures (7). With this in mind, the rationale for the development of new antiepileptogenesis strategies, involving epilepsy prevention and seizure reduction (8, 9) might start with a better understanding of the pathophysiological mechanisms underlying epileptogenesis. In this review, (1) we describe the main histopathological findings of hippocampal sclerosis and their effect on the process of epileptogenesis, (2) we focus and explore the contribution of mossy fiber sprouting (MFS) to seizure generation and, (3) we discuss the possible role of MFS as a therapeutic target in MTLE.

## MTLE and histopathological features in hippocampus

Three major phases are involved in MTLE (9, 10): (i) acute injury (e.g., *Status epilepticus*, prolonged and complex febrile seizure, or traumatic brain injury); (ii) a latent phase; and (iii) a chronic phase, characterized by spontaneous recurrent seizures. The development of an epileptic condition and/or the progression of epilepsy is called epileptogenesis (11, 12). The hippocampal formation is highly susceptible to epileptic activity (13, 14) and is the site of seizure initiation in patients with MTLE (15–17). Clinical and experimental evidence demonstrated that the hippocampus plays a significant role in the pathogenesis of MTLE. In rodents (18–20) and non-human primate (21) models of temporal lobe epilepsy, *status epilepticus* (SE) induction results in hippocampal sclerosis, characterized by selective neuronal loss (22–26), astrogliosis (27–29), and inflammation (30–32). Within hours to days after the initial injury, hippocampal histopathological features include apoptosis (24, 33–36), dentate gyrus neurogenesis (37–42), the production of ectopic granule cells (43–47), and basal dendrites (48–51). Synaptic reorganization (52–54) and granule cell dispersion (55–58) are late features and their appearance may coincide with the onset of spontaneous seizures (59).

In humans, mortality associated with SE may be as high as 30% due to widespread neuronal damage (4) which is sparse after a spontaneous seizure (60). Similarly, in various animal models of TLE, cell death/damage or apoptosis markers were described along the different time lines of disease progression suggesting that, at least in SE models, the precipitating injury causes massive cell loss, while a spontaneous seizure leads to a variable level of injury (22, 36, 61, 62). However, whether hippocampal neuronal cell loss is the cause or consequence of a seizure, is a topic of discussion in epilepsy research [for review, see (63)]. Despite the reported controversies, a major challenge is to understand how the precipitating injury can produce long-lasting changes in neuronal circuitry and excitability. Different types of injuries may lead to epileptogenesis, sharing underlying mechanisms. Thus, the identification of common components may prevent the abnormal reorganization that transforms a normal brain into an epileptic brain. The search for such epileptogenesis pathways may provide the foundation for the development of novel antiepileptogenic and perhaps, preventive therapies.

There are two types of TLE: one that involves the mesial or internal structures of the temporal lobe; and one called neocortical temporal lobe epilepsy, which involves the outer portion of the temporal lobe. The most common is mesial temporal lobe epilepsy (MTLE), characterized on a pathological level by hippocampal sclerosis (HS) (7). HS is present in 30–45% of all epilepsy syndromes, while it is present in 56% of MTLE [for review, see (64)]. Several schemes have been proposed to classify subtypes of HS, mainly based on the subfield distribution, as well as the extent of hippocampal neuronal loss and gliosis, but a recent consensus classification system, validated by the neuropathology taskforce of the International League Against Epilepsy (ILAE), incorporated aspects of all previous schemes (65). This recent classification does not incorporate other frequent alterations as MFS and interneuron changes. ILAE type 1 HS (moderate to extensive neuronal loss and gliosis in CA1 > CA4, CA3 with sparing of CA2) has the highest seizure-free rate (70–85%) post-resective surgery at 2 years and is commonly associated with febrile seizures (50–76%) (64). About 30–40% of TLE patients present normal appearing hippocampi on magnetic resonance imaging (MRI) studies, with no or only mild neuronal loss on histological examination (66). Patients with no neuronal loss and no gliosis have a poorer postsurgical seizure-free outcome (42–58%) (64). With or without hippocampal sclerosis, patients investigated with MRI have shown that structural damage is not limited to the temporal lobe, with extension of damage to regions such as in the entorhinal cortex, parahippocampal, and fusiform gyrus, thalamus, basal ganglia, amygdala, and frontal and parietal lobe (66–68).

### Hippocampal sclerosis

Hippocampal sclerosis is characterized by intensive gliosis combined with a selective loss of neurons in the hippocampal formation. In the dentate gyrus, the loss of hilar inhibitory interneurons that project to the distal dendrites of granule cells, was hypothesized to produce a direct disinhibitory effect on granule cells (69–71). The loss of excitatory hilar mossy cells was also hypothesized to cause granule cell hyperexcitability (72). This would be indirectly related to the decreased excitation of surviving inhibitory basket cells, which are normally excited by mossy cells. Recently, these hypotheses were re-evaluated by using a transgenic mouse line with toxin-mediated mossy cell ablation. Using these mutant animals, investigators demonstrated that extensive ablation of mossy cells caused granule cell hyperexcitability, although the lack of mossy cells *per se* appeared insufficient to cause clinical epilepsy (73). Neuronal cell loss and gliosis affect other components of the hippocampus such as CA1 and CA3 pyramidal cells, in particular the loss of GABAergic interneurons (62), as well as of the limbic system, the amygdala, entorhinal, or perirhinal cortices (23, 61, 74). This phenomenon is also thought to contribute to the increased excitability of the epileptic hippocampus, possibly resulting in additional cell loss. Although multiple factors might be implicated in the genesis of hippocampal sclerosis, it is still not clear why some individuals are more likely to develop hippocampal sclerosis than others (7).

### Astrogliosis

Astrogliosis is another prominent feature of epileptic foci evident in up to 90% of surgically resected epileptic hippocampi (75, 76). As shown in the kindling model, it may play a causal role in the development of seizures and the persistence of seizure disorders (28). Experimental evidence shows that reactive astrocytes are able to secrete molecules with pro-synaptogenic effects [for review, see (77)] that may support the observed neo-synaptogenesis during the latent period in models of epilepsy (78, 79). The expression of the astroglial-derived synaptogenic molecule thrombospondin 1 (TSP-1) is augmented in astrocytes following brain injury and seizures (80). While TSP-1 has the ability to induce excitatory synapses (81), gabapentin, an antagonist of TSP-1 receptor α2δ1, reduces the incidence of epileptiform discharge, and has neuroprotective effects probably achieved by suppressing the formation of excitatory synapses after trauma (77, 82).

### Mossy fiber sprouting

**Figure 1:**
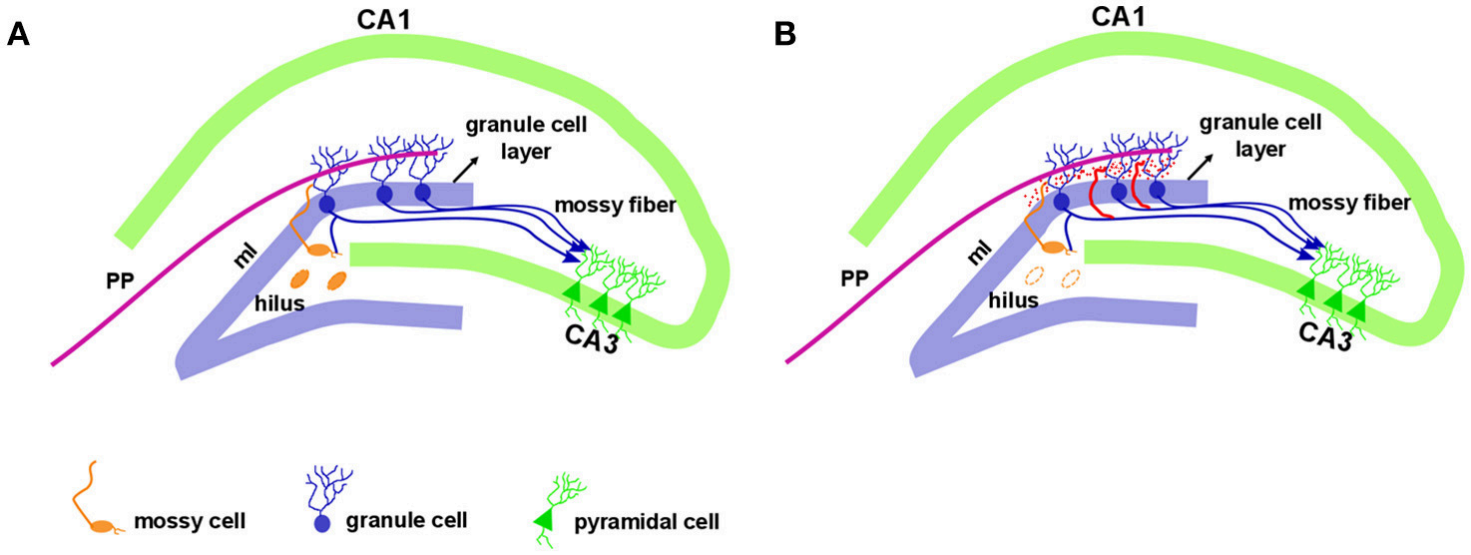
Hippocampal formation in the normal and epileptic brain. The dentate gyrus granule cell layer is densely packed with small diameter cell bodies named granule cells. Just above the granule cell layer is the molecular layer (ml) that is considered cell-free, as it contains the apical dendrites of granule cells. The outer molecular layer receives entorhinal cortex information via a performant pathway (PP). Granule cell axons, named mossy fibers, extend to the hilus with projection to the mossy cells and CA3 pyramidal neurons. The mossy cell axons project to contralateral granule cell dendrites in the inner molecular layer **(A)**. In the epileptic hippocampus, with the loss of mossy fibers target in the hilus, the granule cell axons sprout and extensively innervate the dentate inner molecular layer of the hippocampus, a phenomenon called mossy fiber sprouting, illustrated in red **(B)**.

In addition to hippocampal sclerosis and astrogliosis, the aberrant sprouting of granule cell axons, known as mossy fiber sprouting (MFS), is a frequent histopathological finding in TLE (83–85). Formation of MFS occurs in two phases: (1) the injury *per se* induces neuronal activity and the release of growth factors (86–88), and (2) the growth and extension of the granule cell axon (89–91). Morphologically, the hippocampal dentate gyrus contains three layers: molecular layer, granule cell layer, and polymorphic layer, also known as the hilus. This well-characterized pattern is conserved across mammalian species. The molecular layer is considered cell-free, as it contains the apical dendrites of granule cells and the excitatory terminals that convey information from either the entorhinal cortex (to the outer molecular layer) or from the commissural projections (to the inner molecular layer). The granule cell layer is densely packed with small diameter cell bodies (granule cells). The granule cell axons (also named mossy fibers) extend to the hilus and project to the excitatory interneurons (mossy cells) and to inhibitory interneurons before running through a narrow area called the *stratum lucidum*, to synapse onto CA3 pyramidal neurons (Figure 1A). Rarely, mossy fibers synapse onto other granule cells (92). The mossy cell axons project to contralateral granule cell dendrites in the inner molecular layer (associational pathway of the dentate gyrus or the commissural pathway in rodents) as well as to inhibitory basket cells, located within the granule cell layer. Thus, an excitatory input generated in the entorhinal cortex reaches the granule cells and extends to mossy cells, which in turn inhibit the granule cells. For a detailed description of hippocampal circuitry see Amaral et al. (93).

**Figure 2:**
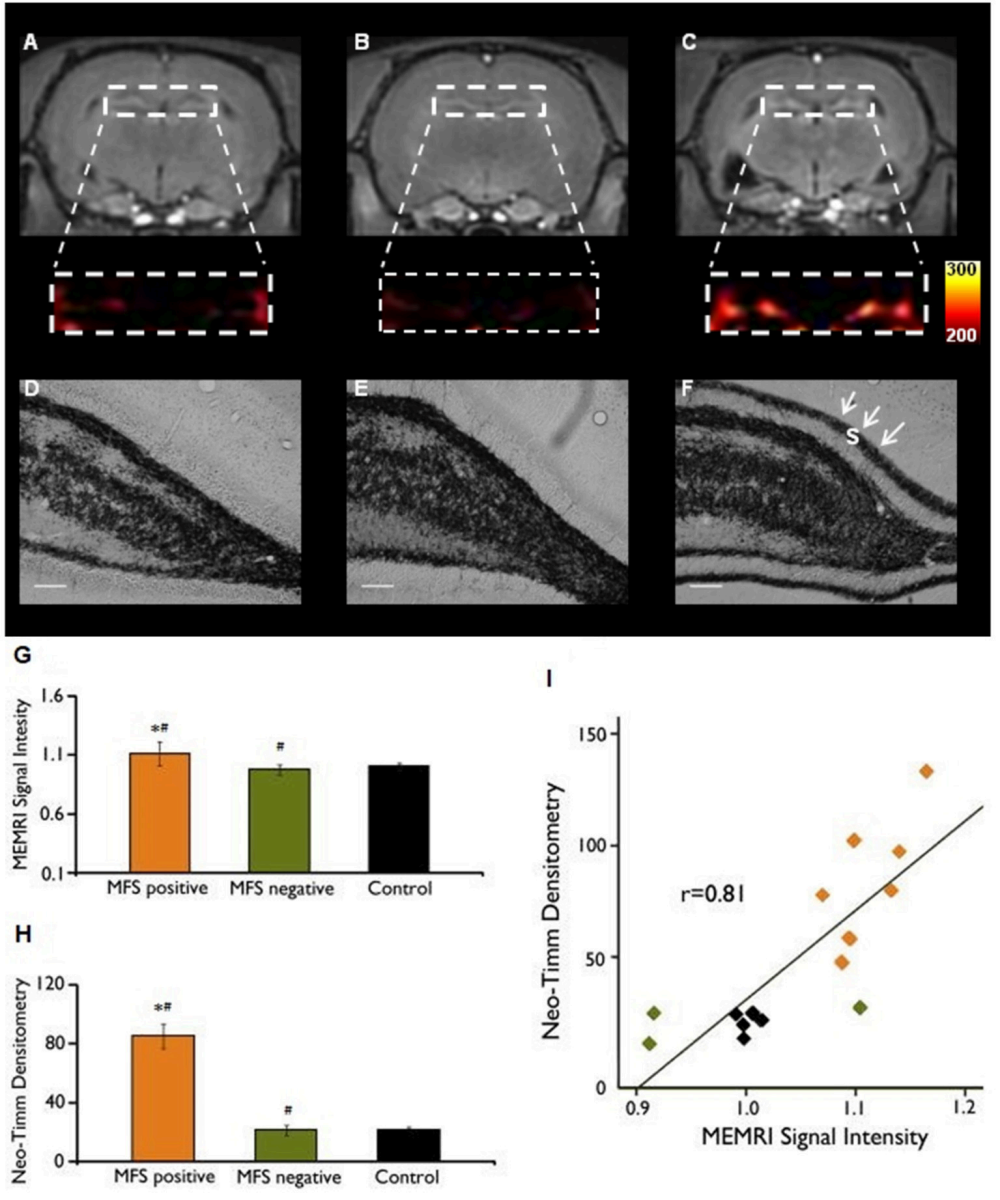
MEMRI and neo-Timm analyses for MFS positive and MFS negative animals. T_1_-weighted Manganese-enhanced MRI (MEMRI) **(A–C)** and neo-Timm staining **(D–F)** in Pilocarpine-treated animals. The protein synthesis inhibitor cycloheximide administered before SE onset was able to reduce the mossy fiber sprouting **(A)** at control levels **(B)** when compared with the Pilocarpine group **(C)**. Differences were identified between MFS positive and MFS negative (^#^*P* < 0.05) and MFS positive and control animals (*P < 0.05) **(G–H)**. Neo-Timm densitometry and MEMRI signal is strongly correlated (*r* = 0.81; *P* < 0,001) in the dispersion graph **(I)**. s: supragranular mossy fiber sprouting (white arrows in **F**). Scale bars = 50 μm. Modified from our previous study (99).

While mossy fibers are hardly seen in the molecular layer of human non-MTLE resected hippocampal tissue, a robust density of mossy fibers containing zinc-rich terminals can be visualized with neo-Timm staining in MTLE specimen (94). Originally described in MTLE patients (94), the mossy fiber sprouting refers to an abnormal and extensive innervation of mossy fibers to the dentate inner molecular layer of the hippocampus (Figure 1B). Similar findings were soon demonstrated after kainate-induced seizures (95), and widely replicated in other animal models [for review, see (96)]. While the neo-Timm staining protocol is considered the gold standard to identify the aberrant zinc terminals in the inner molecular layer, in many animal models of TLE (red dots in the Figure 1B), recent imaging data has revealed that MFS can be tracked *in vivo* in longitudinal studies, by means of the manganese-enhanced signal in T1-weigthed images (97–100), allowing many possible correlational studies (Figure 2).

The aberrant MFS initiates with the formation of new sprouts from mossy fibers within the dentate hilus, which is likely triggered by an injury-related increase in neuronal activity and the upregulation of brain-derived neurotrophic factor (86–88). Two distinctive mechanisms were proposed to contribute to the extension of mossy fiber sprouts to the inner molecular layer: the vacancy of synaptic sites (101) in granule cell proximal dendrites, caused by hilar cell loss after injury (70) and the downregulation of chemorepellents, such as Sema3A (102). Sema3A, normally secreted by entorhinal axons projecting to the dentate molecular layer, interacts via a receptor complex composed of neuropinlin-1 and plexinA present in dendrites of adult granular, hilar and pyramidal cells, suggesting that this signaling pathway is active in the hippocampal formation [for review see (103)], but is lost after status epilepticus. This suggests that the downregulation of chemorepellents, such as Sema3A, may act as a molecular element that facilitates the formation of recurrent projections of mossy fibers into the inner dentate molecular layer after status.

Originally described as a functional recurrent excitatory projection to dentate granule cells (95), caused by the loss of mossy fiber target cells in CA4 and CA3, the origin (104) and the role of MFS in MTLE has been disputed (105, 106). Hilar cell loss is an early finding in experimental models of TLE (107). However, there is no clear evidence that it is the specific loss of mossy cells and not the loss of other hilar interneurons, that triggers the MFS (108). Nowadays the topic of whether MFS is “epileptogenic” or “restorative” remains controversial. Although intrahippocampal circuit reorganization may be a cause of hippocampal epileptiform activity, recent studies indicate that sprouting is not necessarily associated with the occurrence of spontaneous seizures (109). The supportive evidence for both hypotheses is discussed below.

## Mossy fiber sprouting: excitatory or inhibitory role in hippocampal circuitry?

The evidence for a pro-epileptogenic role of MFS includes its presence in about 60% of patients with mesial temporal lobe epilepsy (94, 110) and animal models of hippocampal epilepsy (59, 111, 112). Re-assessed in the 1990's (113), the “mossy fiber sprouting hypothesis” holds that the increased excitability of dentate granule cells is the consequence of a pathological rearrangement of neuronal circuitry on which the excitatory granule cells innervate themselves, building up recurrent excitatory circuits. This hypothesis was supported by several lines of evidence, some described below. Electron microscopic studies show that sprouted mossy fiber terminals form asymmetric (excitatory) contacts with dendritic spines of granule cells (83). Electrophysiological evidence was obtained with perforant pathway stimulation (PPS). A single PPS in hippocampal slices of normal rats was able to produce an excitatory postsynaptic potential (EPSP) and a population action potential in the granule cells. If a second PPS is triggered 40 ms later, it evoked the EPSP but not the population action potential, possibly due to an inhibitory recurrent activation—defining the concept of a “gate” role for dentate granule cell layer. When the same experimental setup was performed in hippocampal slices of kainate-treated rats, the second stimulation evoked multiple populations of action potentials, indicating that granule cells became disinhibited and thus, hyperexcitable. These results were correlated with the presence of robust MFS in these slices, as revealed by Timm staining (95), suggesting that the aberrant sprouting of mossy fibers into the molecular layer could be associated with the loss of the dentate “gate.” Similar results were obtained in slices from kainate-treated animals, when antidromic stimulation of granule cells provoked seizure-like bursts of action potentials (111, 112). Important evidence of the pro- epileptogenic role of MFS was demonstrated in the kindling model, in which the density of sprouting increases with the number of induced seizures (114).

Aberrant MFS positively correlates with mossy cell loss in MTLE patients (115) and animal models (45, 116) but is it necessary and sufficient to generate seizures? Arguing against that view, evidence shows that MFS can be induced experimentally without seizures, after long-term potentiation (117), lesion of the perforant pathway (118, 119), or genetic mutation (120). Similarly, after electrical stimulation of the amygdala, some animals develop seizures but not MFS (106). In the prolonged febrile seizures model, MFS is well-developed 3 months after the initial injury, despite the absence of significant cell loss or increased dentate neurogenesis (121). In both the pilocarpine and kainate models, the presence and the intensity of MFS are positively correlated with higher T_2_ relaxation time values, number of spontaneous seizures, and the degree of cell loss in the granule cell layer, CA1 and CA3 pyramidal cell layer, but not in the hilus (116). This data is consistent with previous reports (122, 123) and indicates that although important, MFS may develop independently of mossy fiber target loss (124), it is present in animals with spontaneous seizures but its presence is not necessarily associated with the occurrence of spontaneous seizures (106).

Some data indicate that MFS is an active phenomenon, possibly a normal adaptive mechanism that becomes pathogenic (125, 126). Evidence that favors the idea that MFS is an active phenomenon came from studies using the mTOR pathway inhibitor, rapamycin. Rapamycin is an Akt (protein kinase B)-mTOR inhibitor, which is related to various neuronal functions, including synaptic plasticity, neurogenesis, and dendritic and axonal plasticity (127, 128). Treatment of epileptic animals with rapamycin for 2 months after SE onset reduced MFS by half. Later evaluations indicated that once the rapamycin treatment ended 2 months after SE, MFS resumed and became fully developed 6 months after SE (126–128). Another Akt inhibitor called perifosine, was used prior to SE induced by kainic acid (KA). Pretreatment with perifosine suppressed the KA-induced neuronal death and MFS, resulting in a decrease in spontaneous seizures (129). These data support the hypothesis that MFS, after the epileptogenesis process, may lead to, but it is not necessary for the formation of recurrent excitatory circuits in dentate granule cells.

Accordingly, although more zinc-containing terminals are seen (by using Timm staining) in animals that have more seizures, the ultrastructural analysis of dentate molecular layer failed to show an increased number of excitatory synapses, favoring the idea that MFS is related to replacement or restoration of lost contacts rather than to increased excitability (125, 130). Other evidence against the excitatory role of MFS has also been suggested in electrophysiological studies. Even though commissural fibers are excitatory and might be predicted to excite granule cells, the activation of this pathway *in vivo* has a predominantly inhibitory effect on granule cells. This is likely because the mossy cell-derived commissural pathway, directly excites inhibitory basket cells (131–133). MFS may re-innervate both basket cells and granule cells, which were found to be disinhibited and hyperexcitable immediately after hilar neuron loss, prior to MFS (84, 134). Another proposed mechanism is dependent on Neuropeptide Y (NPY) which is highly expressed in mossy fibers of pilocarpine-treated rats. When spontaneously released from the recurrent mossy fiber terminals, NPY reduces glutamate release by presynaptic activation of Y_2_ receptors, depressing the epileptiform activity of granule cells dependent on the recurrent innervation (135, 136). Thus, the imbalance between excitatory/inhibitory inputs is believed to be the underlying mechanism of the MFS action. Accordingly, optogenetic approaches have been used to induce excitation of GABAergic interneurons and thus, seizure suppression (137–139). However, recent evidence argues for a possible “excitatory” role of GABAergic cells, depending on the context in epileptic circuitry. The dual roles of GABAergic interneurons was recently addressed, demonstrating that these neurons can (1) excite postsynaptic neurons due to the elevated reversal potential of Cl^−^ in the postsynaptic cells; (2) be GABA-depleted with continuous activity; (3) synchronize network activity during seizures; and (4) inhibit other interneurons, causing disinhibition of principal neurons and network excitability (140).

Although MFS is a common finding in MTLE, not all patients with spontaneous seizures develop MFS (141, 142), a finding that is corroborated in some animal models (99, 143, 144). As a result, MFS would not be necessary for triggering or maintaining hippocampal hyperexcitability. Favoring this idea, cycloheximide, a protein synthesis inhibitor, when used at SE onset did not interfere with induced and spontaneous seizures, but suppressed MFS in the pilocarpine and KA models (101, 143–145), as confirmed by electron microscopy (130). The mechanisms underlying such suppression remain unclear, but may be associated with a neuroprotection of hilar cells (38). Although complete suppression of MFS by cycloheximide treatment was not confirmed by other laboratories, their results indeed demonstrated a reduction of MFS (146). Regardless of whether MFS is suppressed or reduced after cycloheximide, these studies demonstrate that MFS can be modulated and does not directly interfere with the occurrence of spontaneous and recurrent seizures.

Thus, MFS is neither pro- nor anti-epileptic and has also been suggested to be an epiphenomenon (147). Adult-born granule cells robustly contribute to MFS and form functional recurrent synapses (148) as do the neonatally born neurons (40, 149). Considering the increased neurogenesis rate after seizures, it is likely that continued neurogenesis results in increased MFS, which is further reinforced by subsequent spontaneous seizures and results in increased dentate excitability. However, it was recently demonstrated that despite the presence of robust morphological MFS from granule cells born after status epilepticus, these synapses were not functionally active, unable to drive recurrent excitation (148).

## Alternative epilepsy treatments with or without effect on mossy fiber sprouting

MTLE is one of the most prevalent forms of refractory symptomatic epilepsy. Despite the effectiveness of pharmacological therapy in controlling seizures in more than two thirds of cases, some patients develop unacceptable pharmacological side effects (150), which makes continuing the search for better treatment options of utmost importance. Although MFS's role in epileptogenesis is not entirely understood, the close association between aberrant MFS and epileptogenesis indicates that therapeutic strategies capable of suppressing MFS into the inner molecular layer, may have potential clinical significance. Thus, MFS may be an important therapeutic target for treatments designed to interfere with, or modulate the axonal guidance system.

When conventional antiepileptic drugs (AEDs) fail to achieve their desired effects and the surgical resection of the focus is not an option, alternative methods are usually explored. Some of these methods include a ketogenic diet (151, 152), vagus nerve stimulation (VNS) (153, 154), deep brain stimulation (DBS) (155–157), cell therapy (158), or new experimental compounds (159, 160). When tested in animal models, the ketogenic diet produced contradictory effects (161, 162), while VNS (163, 164), and DBS (165, 166) led to a reduction in hippocampal excitability and/or spontaneous seizures (165, 166). VNS and DBS are surgical alternative procedures for patients who are not responsive to conventional AEDs and are not candidates for surgical resections (e.g., due to multiple seizure foci or foci in eloquent regions).

### Vagus nerve stimulation

One possible underlying mechanism for the effects of VNS, is the increase of extracellular norepinephrine concentrations (167) in the hippocampus (168), amygdala (169), and cortex (168). VNS could inhibit seizure activity in PTZ-kindled rats (170) and delayed the development of seizures in cats after KA treatment (171). Recently it was demonstrated that intermittent VNS is able to increase the expression of the fibroblast growth factor (FGF) and the brain-derived neurotrophic factor (BDNF) in the hippocampus and cortex of rats (172, 173); to increase proliferation in the dentate gyrus (174). There is however, no evidence that VNS affects the MFS.

### Deep brain stimulation

Deep brain stimulation of the anterior thalamic nucleus (AN) has been approved for the treatment of medically-refractory partial epilepsy (155). In preclinical models, AN DBS was shown to reduce the frequency of seizures (165) and increase the latency for SE (175, 176). The anticonvulsant effects of AN stimulation were also demonstrated prior to pilocarpine treatment, resulting in an increased latency for seizures and SE (175, 176). In the long-term, AN DBS during SE, results in an increased latency for the development of chronic recurrent seizures and neuroprotection in the dentate gyrus and CA1 regions (158). The neuroprotective role of AN DBS during status, or in the chronic phase, may occur due to a reduction in apoptosis and neuroinflammation (33), as well as hippocampal excitability (166) and increases hippocampal adenosine levels (166), suggesting that adenosine is involved with neuroprotection, in this model. Similar results were observed when DBS was applied to other brain targets (e.g., hippocampus) (159). Despite its seizure-modulating actions, AN DBS does not alter neo-Timm expression in the pilocarpine model of epilepsy (177).

### Cell transplantation therapy

Recent studies have focused on cell transplantation, as an attempt to replace neuronal loss in various hippocampal subfields and/or to explore its potential to release disease-modifying substances. The effects of transplants on spontaneous seizure suppression, are promising. On one hand, there was no reduction in the percentage of rats that developed spontaneous seizures, but transplanted rats displayed fewer spontaneous seizures than sham-transplanted controls after KA treatment (178). Using cell therapy, Bortolotto and colleagues reduced the number of spontaneous seizures, but there was no difference in the kindling susceptibility following grafting (179). In another recent study, a long-term reduction in the number of spontaneous seizures was found in mice after the intra hippocampal transplantation of progenitor cells from embryonic medial eminence after pilocarpine-induced SE (180). Endogenous cell transplantation can be genetically manipulated to affect and modify disease progression. Important results were found using GABAergic progenitor cells. These grafts of GABAergic neurons were able to suppress seizures by enhancing synaptic inhibition in a chronic epileptic animal model of pilocarpine in mice (180–182). Therefore, despite a few controversial studies, there is some work showing a great therapeutic potential for the transplanted cells.

Human fetal stem cell treatment was assessed in the pilocarpine model of TLE to reduce seizures. This treatment showed extensive migration of the implanted cells around the injection site, along with differentiation (24% produced GABA); increased glial cell-derived neurotrophic factor (GDNF) levels, but did not reverse MFS (183). Opposing results were recently demonstrated following the intravenous infusion of mesenchymal stem cells from rat bone marrow, which was associated with the neuroprotection, reduction of cognitive deficits and suppression of MFS (184). The authors concluded that grafts might reduce epileptogenesis through the suppression of aberrant MFS (184). Grafts of CA3 fetal cells enriched with FGF-2 and BDNF exhibit robust integration and inhibit the abnormal MFS in the injured hippocampus (185). CA3 cell grafts transplanted into the injured rat hippocampus 4 or 45 days after KA, dramatically reduced the extent of aberrant MFS (~70%). This shows that such techniques may be promising for partially restoring hippocampal pathology after damage and the release of substances that could modulate and interfere with MFS formation and axonal guidance.

Recently, a group of epilepsy resistant patients was treated with AED supplemented with a single intravenous administration of undifferentiated autologous mesenchymal stem cells. These patients either achieved remission (no seizures for 1 year and more) or became respondent to AEDs (158) indicating that stem cell treatment may be promising for the treatment of pharmacoresistant patients.

### Other experimental therapies

MFS was prevented after repetitive administration of nicardipine, an L-type Ca^++^ channel blocker that also ameliorated the cognitive deterioration but had no anticonvulsant action against pilocarpine seizures (159). A list of a few relevant studies with different subjects, models and treatments for MTLE is listed in Table 1. In this table we listed studies that had success in suppressing both the MFS and spontaneous seizures (184, 186, 189); suppressed MFS but had no effect on spontaneous seizures (101, 109, 129, 143, 144, 187, 188) or suppressed seizures but had no effect on MFS (183). Among these studies, systemically infused mesenchymal stem cells (MSCs) suppressed aberrant MFS in the hippocampus in the lithium-pilocarpine injection model (184). However, the use of human neural stem/progenitor cells (huNSPCs) in the pilocarpine model suppressed seizures, but did not reverse MFS (183). Based on that, cellular therapy can be effective in the remission of spontaneous recurrent seizures, which could also be verified in studies with patients (158), but is not always effective in suppressing MFS. Some therapeutic compounds such as resveratrol treatment have the potential for reducing the intensity of injury-chronic epilepsy (192) by decreasing the frequency of spontaneous seizures, protecting against kainate-induced neuronal cell death in the CA1 and CA3 hippocampus and suppressing MFS (190). However, rapamycin and curcumin treatments did not change epileptogenesis (191). Rapamycin, which was effective in suppressing seizures, as long as its blood levels were sufficiently high, had no effects on MFS in electrical post-SE model. According to the authors, curcumin's lack of effect was possibly because it did not reach the brain at adequate levels (191).

**Table 1:** Treatment effects on seizure frequency and MFS.

**Study**	**Subject/Model**	**Treatment**	**Main Effects**
Chen et al. (186)	Pilocarpine and pentylenetetrazole kindling model	Overexpression of repulsive guidance molecule (RGMa) in the hippocampus	Reduced spontaneous seizures and MFS.
Heng et al. (109)	Pilocarpine	Rapamycin	Blocked MFS but not seizures.
Buckmaster and Lew (187)	Pilocarpine	Rapamycin	No effect on spontaneous seizures but suppressed MFS.
Buckmaster et al. (188)	Pilocarpine	Rapamycin	Reduced MFS, which was restored after the end of the treatment.
Zhu et al. (129)	Kainic acid	Perifosine	Suppressed neuronal death and MFS.
Li et al. (189)	Perforant path kindling	Reo3Y, ligand of the p65/p95 receptor	Suppressed perforant path kindling, MFS, and hilar changes.
Longo et al. (143–145)	Pilocarpine and kainic acid	Cycloheximide	No effect on spontaneous seizures but suppressed MFS.
Fukumura et al. (184)	Pilocarpine	Mesenchymal stem cells	Suppressed MFS, reduced seizures and induced neuroprotection.
Rao et al. (185)	Kainic acid	Grafts of CA3 fetal cells enriched with FGF-2 and BDNF	Dramatically reduce the extent of the aberrant MFS (~70%).
Lee et al. (183)	Pilocarpine	Human fetal stem cells	Suppress seizures, but had no effect on MFS.
Wu et al. (190)	Kainic acid	Resveratrol	Decreased the frequency of seizures, protect against neuronal death, and suppress MFS.
Drion et al. (191)	Electrically-induced SE	Rapamycin and curcumin	Suppress seizures depending on rapamycin blood levels, but no effect on MFS. Curcumin treatment had no effect on chronic seizures.

## Conclusion

**Figure 3:**
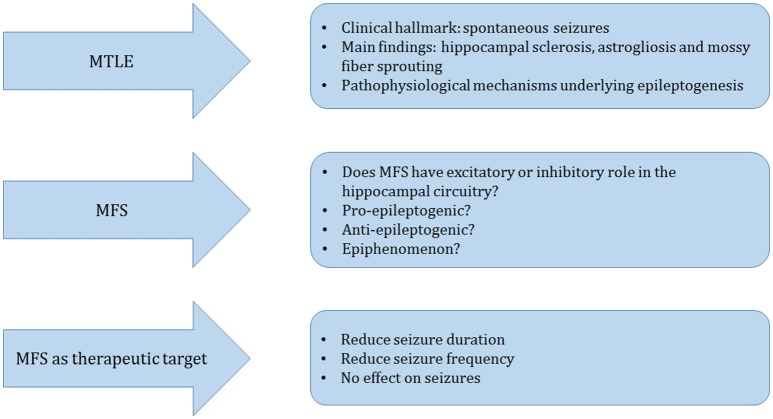
Schematic representation of the main topics of this manuscript. Mesial temporal lobe epilepsy (MTLE) is clinically hallmarked by spontaneous seizures. The main pathological findings in the surgically resected hippocampus of patients and animal models of TLE are hippocampal sclerosis, astrogliosis, and mossy fiber sprouting (MFS), which are frequently studied to understand the mechanisms that may underlie epileptogenesis. We discussed three main different perspectives: Does MFS have a pro- or anti-epileptogenic role or is it an epiphenomenon? We finalize our review with a series of current alternative therapeutic approaches to reduce seizures and excitability that may affect or not the MFS.

In this article we reviewed MFS, as a pathological substrate for MTLE (Figure 3). Other morphological alterations include hippocampal sclerosis, astrogliosis, neurogenesis, cell dispersion to name a few. The contributing role of each one of these malformations for the development of epileptogenesis is not clearly understood. Although MFS is a frequent finding in MTLE it is not necessarily present. Although MFS makes recurrent excitatory circuits, these are likely not sufficient to generate seizures. Based on different strategies such as, protein synthesis inhibitors, calcium channel blockers, stem cell therapy, and rapamycin studies, we learned that MFS is an active process that can be manipulated, but has little or inconsistent effects on seizure suppression Thus, we conclude that MFS is related to replacement or restoration of lost synaptic contacts, rather than to increased excitability in hippocampal circuitry.

## Author contributions

CC and JM worked on the literature search and prepared the first draft of the manuscript. CH and IN participated in the writing process and LC established the manuscript structure, coordinated, and participated in the revision writing.

### Conflict of interest statement

The authors declare that the research was conducted in the absence of any commercial or financial relationships that could be construed as a potential conflict of interest.
